# Femoral neck fracture during physical therapy following surface replacement arthroplasty: a preventable complication? A case report

**DOI:** 10.1186/1754-9493-4-3

**Published:** 2010-02-04

**Authors:** Timothy R Judkins, Michael R Dayton

**Affiliations:** 1 Department of Orthopaedics, University of Colorado Denver, Denver, CO, USA

## Abstract

This case report describes two cases of peri-prosthetic fracture during physical therapy in patients who underwent a hip resurfacing, or surface replacement arthroplasty. The fractures occurred with forceful passive combined flexion and external rotation. Functional results were ultimately obtained in both cases, requiring conversion to total hip arthroplasty. Recognizing patient risk factors and cautioning therapists about the possibility of fracture may have prevented these complications.

## Background

Hip surface replacement arthroplasty (SRA) is an increasingly popular option for treating degenerative hip disease, particularly in the young and active patient [1]. Compared to total hip arthroplasty (THA), advantages include bone conservation and stable range of motion with potentially less risk for dislocation [2].

Common complications of hip SRA include femoral neck fracture, avascular necrosis (AVN) and increased levels of metal ions in the blood [3]. Of known complications, fracture of the femoral neck is well documented, with an incidence in one series of 1.46% (1.91% women and 0.98% men) [4]. Technical factors during surgery may increase the risk of fracture. The most important of these are implant varus alignment and femoral neck notching [4]. Additionally, femoral neck fracture may increase as a function of elevated body mass index (BMI). Regardless of specific risk factor, careful patient selection is imperative when recommending hip SRA due to the increased risk of fractures in females, in those with relative Osteopenia or BMI approaching obesity.

The present report describes two patients that sustained femoral neck fracture during post-operative physical therapy passive range of motion. The fracture was attributed to passive motion maneuvers for both cases, in which the therapist used force to advance each patient's hip into flexion and external rotation.

## Case Report 1

A 59-year-old female presented with a five-year history of progressive right hip pain that radiated to her groin. Her past medical history was significant for borderline diabetes mellitus and hypertension. Past surgical history included bilateral knee arthroscopy. On physical exam this was a Caucasian female with a BMI of 24. Her right hip had limited range of motion to 0-85 degrees of flexion, no internal rotation, and 15 degrees of external rotation. Radiographs demonstrated degenerative joint disease of the right hip, with decreased cartilage space and marginal osteophytes.

The patient elected to undergo hip SRA. This was done through a posterior approach, using a 50 mm cemented femoral component and 56 mm acetabular cup (Smith & Nephew Orthopaedics, Ltd, Bromsgrove, United Kingdom). Simplex-P tobramycin impregnated cement was used for femoral component fixation; the acetabular component was non-cemented. There were no intraoperative complications, and the patient was discharged to home after an uneventful recovery period in the hospital with strict posterior hip precautions (limiting flexion, adduction, and internal rotation) and home physical therapy 2-3 times per week. The patient was allowed immediate weight bearing as tolerated post-operatively, with assistive walker or crutches. Stair ascent and descent was allowed with crutches. Walker and crutches were weaned to a cane by 3 weeks under supervision of a visiting home physical therapist.

**Figure 1:**
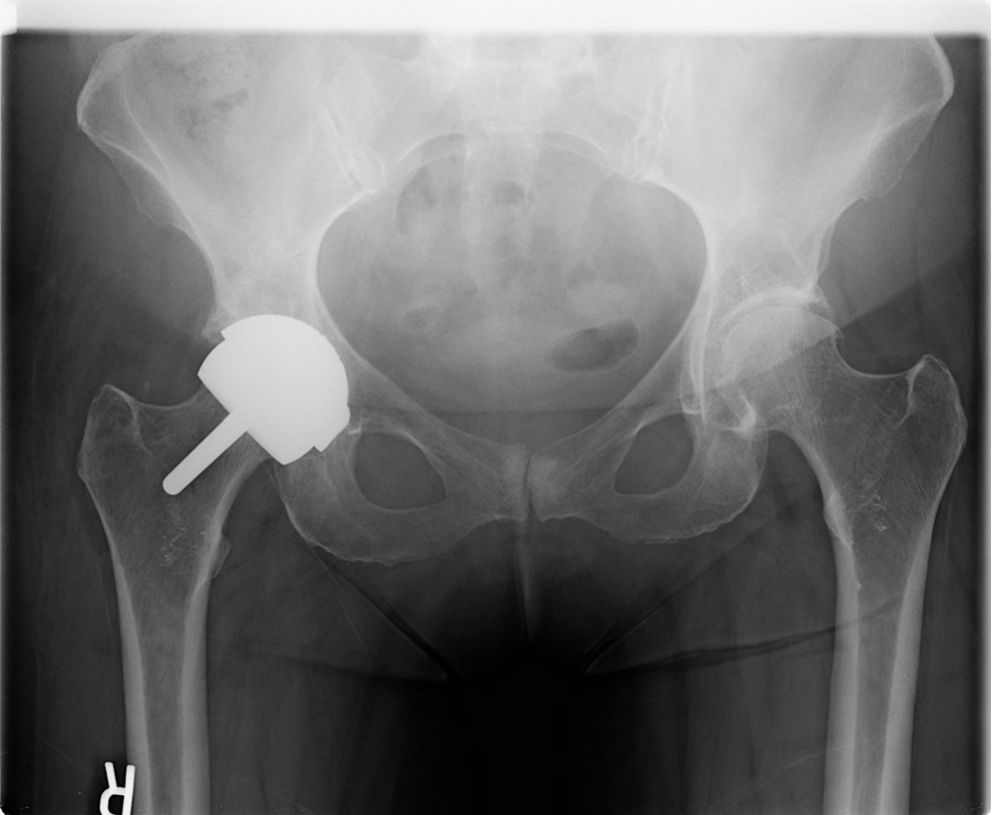
**Case 1: Six weeks after SRA with satisfactory positioning**.

At six-week follow up visit, the patient reported no pain and was no longer using assistive walking devices. Radiographs at that time showed satisfactory placement with no evidence of fracture, migration, or loosening (Figure 1). She was then allowed to discontinue her posterior hip precautions and referred to an outpatient physical therapist for continued outpatient strengthening and range of motion rehabilitation.

**Figure 2:**
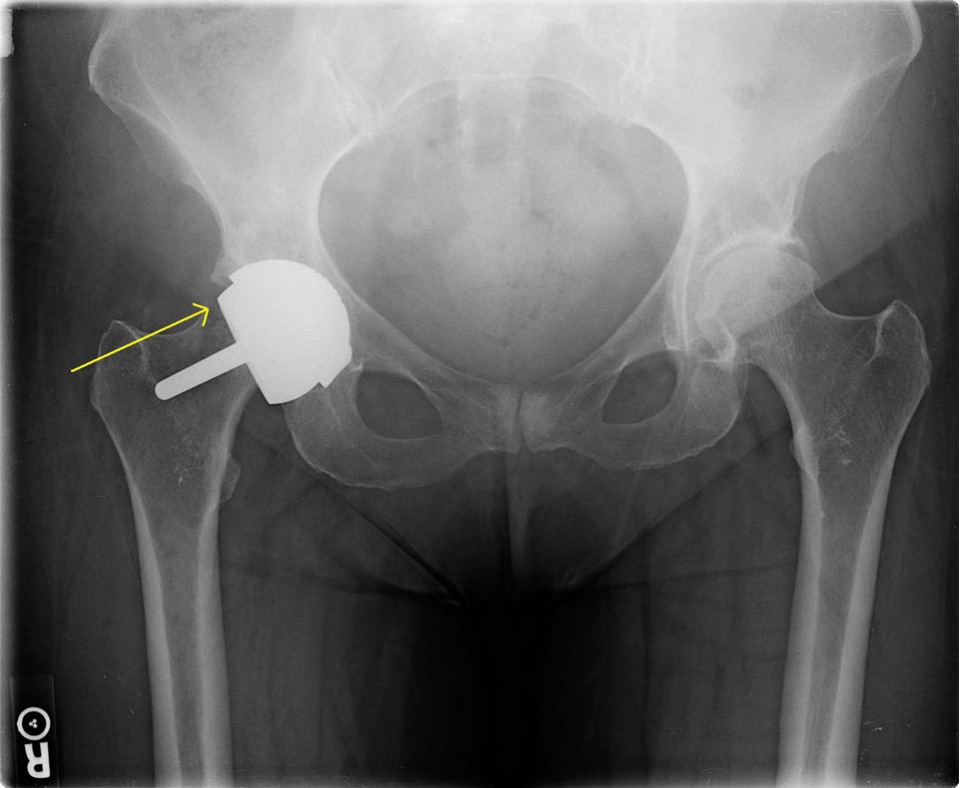
**Case 1: After femoral neck fracture during physical therapy**.

During a session of outpatient physical therapy in post-operative week 7, the physical therapist was performing passive forced combined flexion and external rotation on the patient. With the knee flexed, the therapist placed a constant laterally directed force on the leg and thigh to bring the hip into an externally rotated position. She reported discomfort with the maneuver, but after this session was able to ambulate with weight bearing as tolerated. The patient experienced severe and progressive anterior groin pain over the next 24 hours, yet continued to ambulate with weight bearing as tolerated. She returned for a follow-up physical therapy session five days later with a perceived limp, and requiring a cane for ambulation. She was sent immediately for radiographs, which demonstrated a fracture at the femoral neck with displacement and varus angulation (Figure 2).

The patient was counseled as to treatment options including limited weight bearing, surgical fixation with limited weight bearing, or revision to femoral stem with immediate weight bearing. The patient chose revision and was taken to the operating room for conversion to total hip arthroplasty (THA). This was done using a compatible femoral component (Smith and Nephew Anthology stem, size 7, with 50 mm femoral head and +4 mm sleeve). The acetabular component was stable and retained.

**Figure 3:**
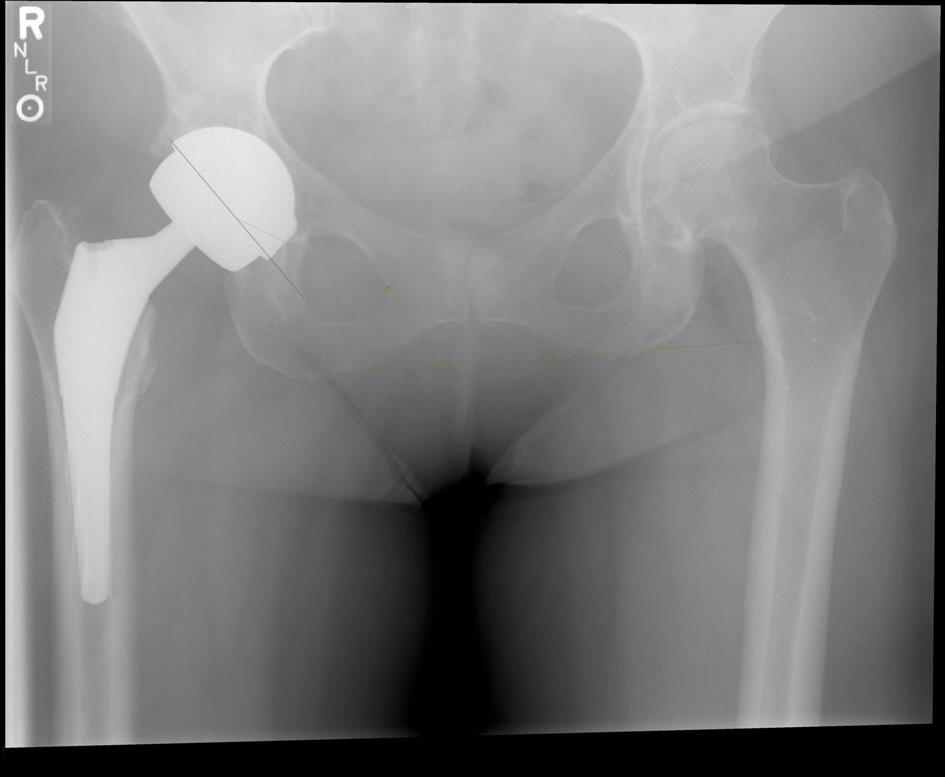
**Case 1: Satisfactory appearance of prosthesis after conversion to THA**.

The patient recovered uneventfully from this surgery with immediate weight bearing and assistive device weaned in an identical manner to her initial procedure. At her post-operative visit six weeks after THA she continued full weight bearing without assistive device and reporting no pain. Radiographs at that time showed satisfactory placement without evidence of migration or loosening (Figure 3).

## Case Report 2

A 51-year-old female presented with right hip pain increasing over several years. She reported difficulty putting her socks and shoes on, and could no longer exercise due to her hip pain. The patient had no other significant past medical or past surgical history. She was also a Caucasian and her BMI was 33. Her physical exam demonstrated painful, limited range of motion. Right hip flexion was 85 degrees, internal rotation less than five degrees, and external rotation less than 10 degrees. Severe degenerative changes were observed on radiographs.

**Figure 4:**
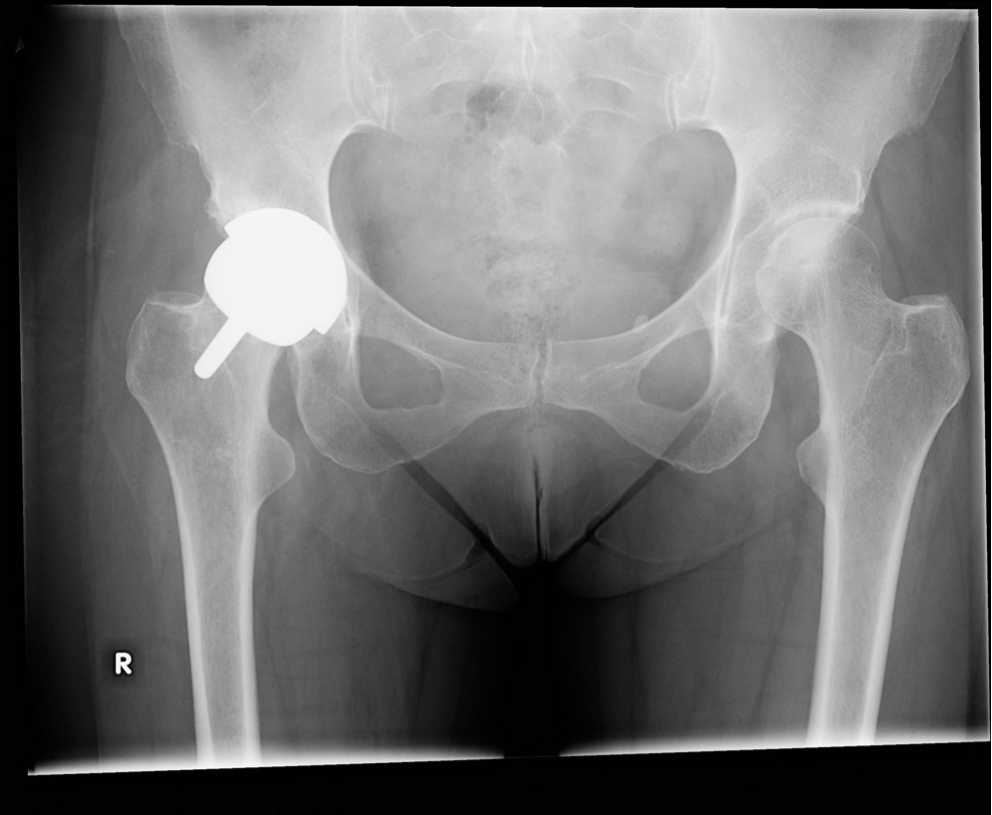
**Case 2: Six weeks post-operatively, with satisfactory placement of the SRA**.

The patient elected to undergo hip SRA in late 2006. A 46 mm femoral head was used along with a 54 mm acetabular cup (Smith & Nephew Orthopaedics, Ltd, Bromsgrove, United Kingdom). Simplex P cement with Tobramycin was used for fixation of the femoral component. After an uneventful hospital stay she was discharged with posterior hip precautions as described in Case #1. Radiographs from the 6-week post-op visit showed satisfactory alignment and no evidence of fracture, loosening, or subsidence (Figure 4). At this time her posterior hip precautions were discontinued, and she began outpatient rehabilitation with a therapist in her community.

She was seen again three months post-op and reported doing very well, able to perform ADLs, and with sufficient functional status to return to full time work. She had no pain, but reported having less than expected range of motion in physical therapy. In particular, she was having difficulty with advancing full external rotation, similar to that noted pre-operatively. This limitation was noted despite consistent flexion, abduction, and external rotation exercises implemented immediately post-operatively.

**Figure 5:**
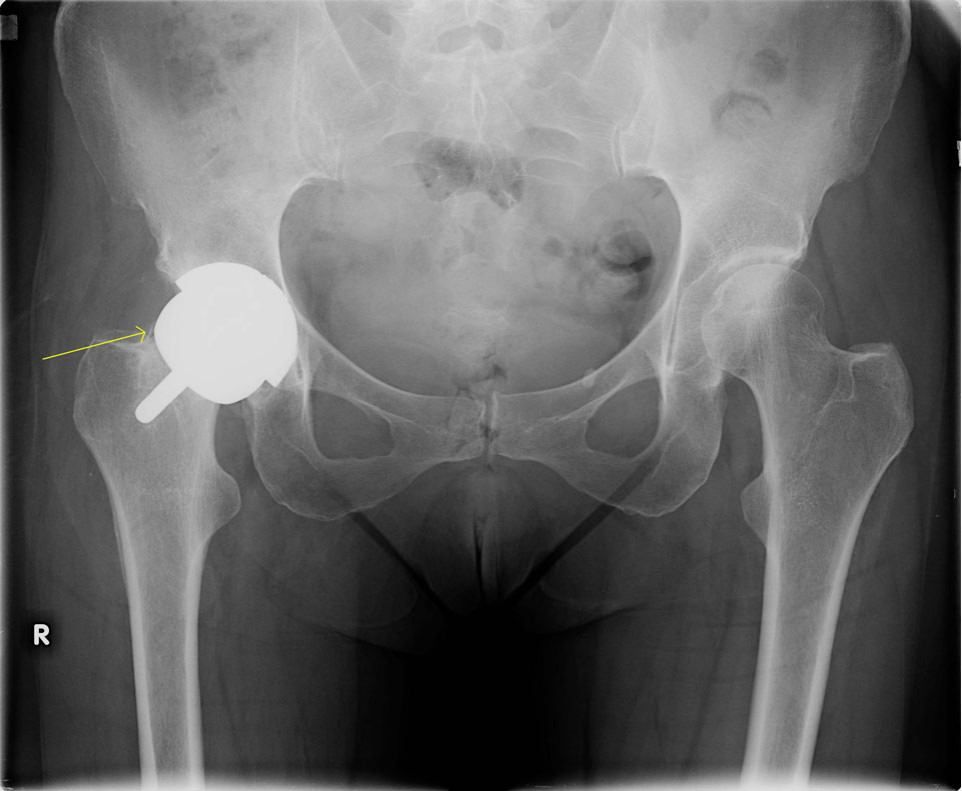
**Case 2: AP pelvis showing non-displaced fracture 20 weeks after SRA**.

Approximately twenty weeks after the hip resurfacing, the patient sustained a painful groin injury during physical therapy. She was undergoing forceful passive external rotation manipulation with her therapist when the injury occurred. The patient started in a "figure of four" position (knee and hip flexed about 90 degrees with the ipsilateral ankle resting on the contralateral thigh). From this position the therapist used steady force to passively flex and externally rotate the hip further. The force that was applied was described as in excess the patient's own active or passive ROM attempts. She reported initial pain after this, but was ambulatory upon leaving the physical therapy clinic. However, the following day she was unable to bear weight and was seen urgently back in clinic, where radiographs confirmed femoral neck fracture (Figure 5).

Treatment options for the peri-prosthetic fracture, including conversion to THA and non-operative treatment with limited weight bearing were presented to the patient. She elected to pursue non-operative treatment, and maintained non-weight bearing for a period of six weeks. After the six weeks she had very little pain. Radiographs at that time showed no significant change in hip SRA placement, but mild lower extremity shortening due to initial positional change of the femoral component. Her weight bearing status was then advanced to fifty percent from 6 to 12 weeks following fracture, then full weight bearing 12 weeks post-fracture.

**Figure 6:**
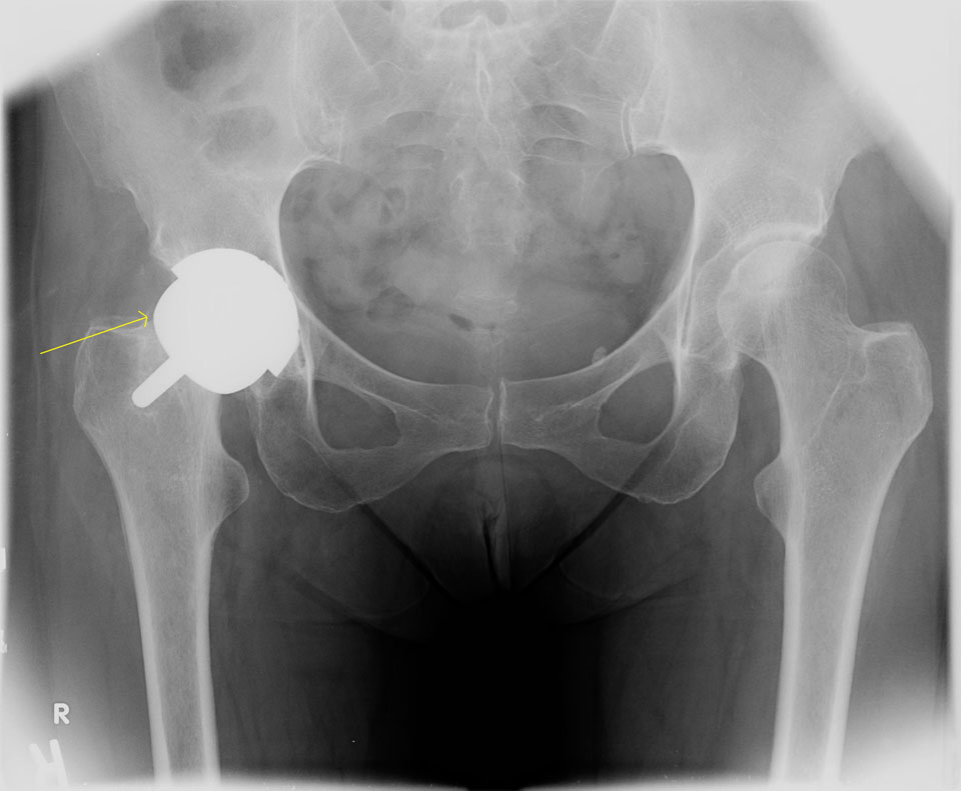
**Case 2: Approximately three months after fracture, treated conservatively**.

After advancing to full weight bearing, she did have a pinching discomfort when ambulating and she used a cane for walking longer distances. Range of motion on examination was 100 degrees of flexion, 30 degrees of external rotation, and 20 degrees of internal rotation, and radiographs at that time showed no further change in implant positioning (Figure 6). No restrictions in weight bearing or ROM were maintained at this point. She was happy with her pain-free status and ROM progress, improved function, and gait. Non-operative treatment continued.

**Figure 7:**
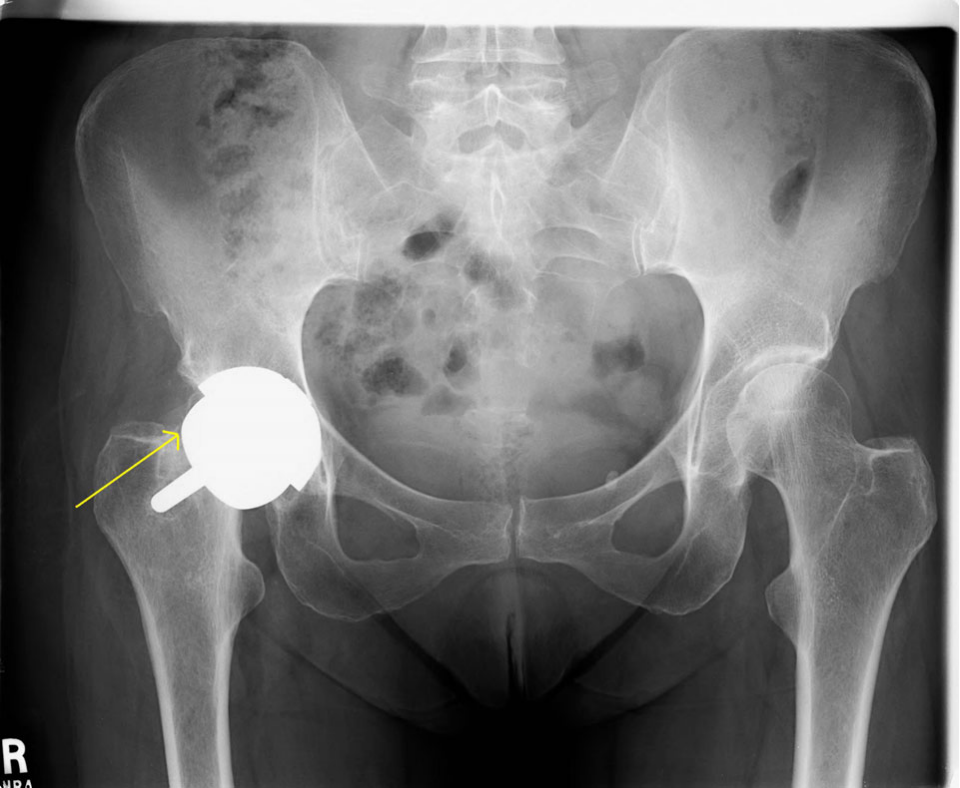
**Case 2: Beyond six months after fracture; evidence of progressive collapse and displacement**.

At nearly one year post-fracture, the patient's pain slowly worsened with more compelling evidence of implant bone collapse on radiographs (Figure 7). The patient ultimately chose to undergo revision to THA at an outside facility in an identical fashion to Case #1 and is now pain free.

## Discussion

Hip SRA is an increasingly popular method for treating hip arthritis, especially in the younger patient population. Hip SRA provides a large metal-on-metal bearing surface that has favorable wear properties and potential for enhanced ROM and lower risk of dislocation. It also has the advantage of preserving bone in the proximal femur for possible future revision to THA. This is particularly important in the younger patient who may likely require revision arthroplasty.

Femoral neck fracture remains one of the primary concerns of hip SRA, with an incidence of 1.91% in women and 0.98% in men [4]. Most cases of femoral neck fracture reported thus far have been related to technical factors, such as varus alignment or notching of the femoral neck. Patient selection is greatly important when deciding between hip SRA and THA. In their review of fifty cases, Shimmin and Back found that older females appear to be at greater risk for femoral neck fracture [4]. This is likely due to the increased incidence of osteoporosis in this age group when compared to males. The presence of large cysts in the femoral head and neck can also preclude the use of hip SRA due to increased risk of fracture [4].

The two cases demonstrated femoral neck fractures that occurred with physical therapy while working on end range-of-motion. While it is not absolutely certain that the fractures occurred during physical therapy, the events surrounding the onset of pain that precipitated evaluation and radiographic study followed the rehabilitation sessions, and were remarkably similar in their described mechanism. Both cases occurred in peri-menopausal females, which placed them at potentially greater risk for fracture due to potentially decreased bone density. Unfortunately, bone densitometry values were not available for these cases. No intraoperative evidence existed, however, of inferior bone quality or intra-osseous cystic changes relative to candidates of similar age, gender, or activity level performed prior to or following these cases. Hence the patients described in this study were deemed appropriate candidates for hip SRA. The fractures occurred at seven and twenty weeks post-operatively, and are thought to have occurred with similar passive range of motion maneuvers (forced flexion and external rotation).

The results of this report demonstrate that specific instructions are mandated when prescribing physical therapy. In the absence of intraoperative notching or varus placement, the patients at greatest risk for fracture are peri-menopausal females, particularly those with pre-existing femoral neck cysts, or small femoral necks. Further, excessive BMI may be an additional risk factor and should be considered in the evaluation of candidacy for hip SRA. While little data exists on the role of BMI as a risk factor relative to bone density and gender, it is likely that excessive weight plays a role in post-operative fracture of the femoral neck. While care is emphasized in patient selection for this population; future fractures may be prevented if these patients and their therapists are cautioned against aggressive passive range-of-motion attempts, especially those including the end-ranges of flexion and external rotation.

## Conclusion

Periprosthetic femoral neck fracture is a well established complication of hip surface replacement arthroplasty. There are numerous documented patient and surgery-related risk factors that increase the risk for fracture. These cases demonstrate a need for physicians and therapists to be cognizant of rehabilitation techniques in order to minimize the risk of this significant complication.

## Consent

Written informed consent was obtained from the patients for publication of this case report and any accompanying images. A copy of the written consent is available for review by the Editor-in-Chief of this journal.

## Abbreviations

SRA: Hip Surface Replacement Arthroplasty; THA: Total Hip Arthroplasty; AVN: Avascular Necrosis.

## Competing interests

One of the authors (MRD) is an employed consultant of Smith Nephew Orthopaedics, a producer of surface hip arthroplasty implants. The authors declare no other competing interests related to this manuscript.

## Authors' contributions

MRD designed the study. TRJ wrote the first draft of the manuscript. Both authors revised and approved the final version of the manuscript.
